# Supplementary material for: Perinatal outcome of growth restricted fetuses with abnormal umbilical artery Doppler waveforms compared to growth restricted fetuses with normal umbilical artery Doppler waveforms at a tertiary referral hospital in urban Ethiopia
Source: PLoS One. 2020 Jun 18;15(6):e0234810. doi: 10.1371/journal.pone.0234810 (PMC7302535; doi:10.1371/journal.pone.0234810)
Supplement: Attachment — Submitted filename: Reviewer response May 10.docx [file pone.0234810.s002.docx]

May 10, 2020

To: PLOS ONE Editor in chief.

Dear Editor in chief.

We would like to thank the reviewers for their thoughtful review of the manuscript. They raise important issues and their inputs are very helpful for improving the manuscript. We agree with almost all their comments and we have revised our manuscript accordingly. We respond below in detail to each of the reviewer’s comments. We hope that you find our responses satisfactory and that the manuscript is now acceptable for publication

Looking forward hearing from you soon

Sincerely,

Lemi B Tolu (MD, Assistant prof of obstetrics and gynecology).

Saint Paul’s Millennium Medical College(SPHMMC)

Department of Obstetrics and Gynecology

Addis Ababa, Ethiopia.

Email: [lemi.belay@gmail.com](mailto:lemi.belay@gmail.com)

**Dear editor and reviewer**

Thanks for thoughtful review of the manuscript. Below is point by point response to raised concerns and how we changed the manuscript according to the comments.

**Editor comments:**

1. Thank you for including your funding statement; "no"

Authors: Dear editor thank you very much, we have corrected as “The authors received no specific funding for this work.”

2. Please amend either the abstract on the online submission form (via Edit Submission) or the abstract in the manuscript so that they are identical.

Authors: Dear editor thank you very much, we have corrected.

**Reviewer #1**

**Title and abstract:**

1) The authors adequately mention the study design. Also, the abstract is adequately structured

**Introduction:**

1) Citations should be place at the end of each line that requires such, not at the end of each paragraph.

2) Introduction must be cut down in half and just stating the essential (disease, rationale, hypothesis, objective)

**Authors:** Dear author thank you very much, almost we rewrite introduction (page 4, lines 69-117)

**Methods:**

1. Please state the study design at the first line of the methods section, then followed by the setting where the study was conducted, then the eligibility criteria.

**Authors:** Dear reviewer corrected as per your recommendation (Page 6-7, lines 128-169).

2. The studies population is supposed to be pregnant women with diagnosis of suspected small-for-gestational-age fetus by estimated fetal weight measurement below the 10th percentile. This population must be adequately defined.

**Authors:** Edited and population well defined, hospital protocol included (Page 6, lines 135-140)

3. Exposures are adequate (normal and abnormal UA).

**Authors:** thank you very much for the comment.

4. After eligibility criteria, variables must be stated in a narrative way, starting with outcomes, then exposures, predictors, potential confounders, effect modifiers. All in a narrative way, without headers.

**Authors**: Dear reviewer comment well taken, and correction made (Page 8, lines 163-169)

5. Please describe the procedure of how pregnant women with abnormal Doppler are handle. What classification do you use? How do you end a pregnancy with abnormal UA Doppler at a certain age?

**Authors:** Dear reviewer thank you very much comment well taken, and protocol of the hospital stated on page 7, lines 135-148.

6. Describe any efforts to address potential sources of bias.

**Authors**: we collected data using structured questionnaire’s, data collectors were not involved in patient care and well trained on the tool, supervisors cross check data collection (Line 232-237).

7. Sample size must be stated before statistical analysis. Which by the way, I want to congratulate the authors for adequately calculating the sample size?

**Authors:** Dear reviewer thank you very much, the manuscript is structed accordingly.

8. Ethical considerations should be reduced to 2 lines maximum.

**Authors**: Comment well taken and addressed (Page 13, 262-264)

9. Statistical methods are ok. The only limitation is How did you adjust for confounders? When describing pregnancy cohorts is almost impossible not to adjust for gestational age at birth as the most important effect modifier.

**Authors**: Dear reviewer thank you very much, we also used multiple regression to control	confounders.

**Results:**

1. For prospective cohorts, please state the number of individuals potentially eligible, examined for eligibility, confirmed eligible, included in the study, completing follow-up, and analyzed. Preferably, use a flow diagram.

**Authors**: Dear reviewer the comment is well taken and addressed (page 13, line 271-276)

2. Tables should be placed at the end of the manuscript and not embedded in the middle of it.

**Authors**: Thank you very much and we moved tables to end of the manuscript.

3. Continuous variables such as maternal age, gestational age... should be stated as mean and standard deviation or median and interquartile range. Continuous variables should not be only categorized, or we miss the idea of the description of the population.

Authors: Dear reviewer gestational age is described in mean and average duration of follow up.

4. Indicate the number of participants with missing data for each variable of interest (if any)

**Authors**: Dear reviewer we didn’t come across missed data (page 13, lines 277-281)

5. State and summarize the follow-up and time of follow. A good way to do so is by describing the mean (and SD) age at diagnosis and the mean age at delivery for each group.

**Authors**: Thanks, comment is well taken and addressed (Page 13, lines 277-281)

6. Thank you for using RRs instead of ORs. As part of the quality assessment, please, describe the adjusted RRs. You can use the Mantel-Haenzel method. The second option is to produce a multiple regression analysis adding the confounders the logistic regression.

**Authors**: Dear reviewer, thank you very much, we also multiple logistic regression.

7. Consider translating relative risk into absolute risk for the given time of the study. This will allow readers to give a meaning idea of the magnitude of the effect.

**Authors:** Dear reviewer thank you very much for the suggestion, but we didn’t think that translating in to absolute risk is as such applicable in the current manuscript.

8. Table 4. It seems clear to me that gestational age is the most important component for adverse outcome. All analyses need to be adjusted for gestational age at birth. Also, those RR with “0” are incorrect. Maybe the analysis was not robust enough. I suggest using bootstrapping to estimate those confidence intervals due to the extremely small sample.

**Authors:** Dear reviewer this is an amazing catch, loved it. Thank you very much, we have corrected it (Table 4)

**Discussion:**

2. Please, discuss more in depth the limitations of the study. Consider the management of women with abnormal UA Doppler.
3. Conclusions must be cut by half at least.

**Authors**: Dear reviewer thank you very much manuscript is modified accordingly (Page 21, lines 388-394 and Page 22, lines 400-404)

**Critical appraisal**

**Authors**: Dear reviewer thank you very much for such input which helped us to improve the manuscript.

**General comments:**

1. It is important to follow the same order as the STROBE tool in a narrative way.
2. Please, add an explanation of possible confounders and how were they handled.
3. Adequately define and explain the selected population (suspected SGA).
4. Use the term suspected small-for-gestational-age for all fetuses below the 10th percentile, and fetal growth restriction for those suspected SGA + abnormal Doppler.
5. Clearly state the hospital’s protocol on how FGR fetuses are handle
6. Please use mean and SD (or median and IQR) for continuous variables
7. Add the gestational age at diagnosis and the gestational age at delivery

**Authors**: Dear reviewer these general comments are addressed in the above explanations.

Minor:

1. Tables should be at the end of the article
2. Use the same font type and number for the whole manuscript. Do not use bigger fonts for the headers

**Authors:** Dear reviewer we have moved tables to the end of manuscript and the manuscript is also prepared according to PLOS ONE requirement.

**Reviewer#2**

**General**

1. Thus, UA Doppler cannot be used as standalone criterion to differentiate IUGR from SGA.

**Author**: Dear reviewer we never said UA is a standalone criterion, but we said its most commonly used. MCA, UA, UV and DV are often used together. Our only aim in the current study is to compare perinatal outcome of abnormal UA to normal UA, we have also included the protocol used in our hospital (Page 7, lines 133-147). Special in low and middle-income countries Umbilical artery is very commonly used for diagnosis and follow up growth restriction.

2. The authors do not provide a complete outline of the current literature in this field and the articles used as references are not complete.

**Authors**: Dear reviewer we have revised the introduction part by including updated information we came across in our search.

**Introduction.**

1. Introduction is very long and must be trimmed

**Authors**: dear reviewer thanks for the comment, we have revised and rewrote introduction accordingly.

**Materials and methods:**

1. The definition for the late and early IUGR fetuses, SGA and non-SGA are required.

Authors: Dear reviewer the comment is well taken and addressed on page 7, lines 135-149.

2. Among the exclusion criteria did they also consider the presence of diabetes, hypertension

**Authors**: Dear reviewer we excluded diabetes, but we included hypertension and did multiple logistic regression to control its confounding effect.

3. More information is required regarding the management of time of delivery, according to the local management guidelines.

**Authors**: Dear reviewer, the comment is well taken and addressed on page 7, lines 135-148.

4. Were fetal well-being tests performed in all cases as standard protocol? please describe the monitoring and management protocol for IUGR

**Authors:** Dear reviewer we have included hospital protocol which includes follow up of fetal wellbeing (Page 7, lines 135-148)

5. Doppler results of the middle cerebral artery and uterine arteries should be added.

**Authors:** Dear reviewer in the hospital we conducted study MCA is done for patients with suspected IUGR but normal UA doppler flow to measure cerebroplacental ratio to see brain sparing which is expected to occur before umbilical artery abnormality, but not considered as universal standard practice. Uterine artery doppler is used for prediction of preeclampsia and growth restriction (predicting uteroplacental insufficiency). The hospital doesn’t use uterine artery for diagnosis or follow up of growth restriction, we didn’t come across literature suggesting such practice otherwise. Dear reviewer that’s why we focus on comparing growth restriction with normal and abnormal umbilical artery. Thanks for understanding us.

**Discussion**

1. It should be clarified why the Doppler values of MCA and uterine artery were not considered for the classification and management of IUGR cases.

**Authors**: Dear reviewer please see the explanation under question number 5.

2. The following references should be added and commented in the discussion.

Authors: Dear reviewer thank you very much we have used the following references in our manuscript from the suggestions.

a. GARDOSI J, MADURASINGHE V, WILLIAMS M, MALIK A, FRANCIS A. Maternal and fetal risk factors for stillbirth: population-based study. BMJ 2013;346: f108.
b. FIGUERAS F, EIXARCH E, GRATACOS E, GARDOSI J. Predictiveness of antenatal umbilical artery Doppler for adverse pregnancy outcome in small-for-gestational-age babies according to customised birthweight centiles: population-based study. Bjog 2008; 115:590-4.
c. ALFIREVIC Z, STAMPALIJA T, GYTE GM. Fetal and umbilical Doppler ultrasound in high-risk pregnancies. Cochrane Database Syst Rev 2010:CD007529.
d. OROS D, FIGUERAS F, CRUZ-MARTINEZ R, MELER E, MUNMANY M, GRATACOS E. Longitudinal changes in uterine, umbilical and fetal cerebral Doppler indices in late-onset small-for-gestational age fetuses. Ultrasound Obstet Gynecol 2011; 37:191-5
e. DOCTOR BA, O'RIORDAN MA, KIRCHNER HL, SHAH D, HACK M. Perinatal correlates and neonatal outcomes of small for gestational age infants born at term gestation. Am J Obstet Gynecol 2001; 185:652-9.

**Reviewer #3**

1. The Introduction provides outdated information and lacks references; i.e. the concepts that UA is the only vessel used for IUGR, or the classification for symmetric and asymmetric IUGR.

**Authors**: Dear reviewer the introduction is rewritten incorporating comments. We didn’t state UA as the only vessel, but we said its commonly used and studied. Special in low and middle-income countries umbilical artery is very commonly used for diagnosis and follow up of fetuses with growth restriction. Thanks for understanding us.

2. I am not sure the sample size calculation is adequate, since they claim it is 150, but usually this would be per study group. Since the proportion of IUGR with abnormal UA Doppler is rare, this would seem logical.

**Authors**: Thank you very much, that’s what we got by calculation as it stated it’s also a rare scenario too.

3. The definition of IUGR is never provided, the authors claim that the diagnosis was by attendings or ObGyn residents. This would be unacceptable due to the variability and lack of expertise, and this population would have to be confirmed or supervised

**Authors**: Dear reviewer the hospital protocol is included in the manuscript which has also definition of IUGR (Page 7, lines 135-	148)

4. The Methods section lacks the information for statistical analysis; Chi squared is only used for comparison of parametric proportions, however the authors present comparisons of medians(range), do not specify if they tested normality and do not describe their analysis adequately.

**Authors**: Dear reviewer we used chi square, cross tabulation and regression.

5. I would suggest the authors redefine their population because their results are very strange; for starters they have a high proportion of abnormal UA Doppler near term than preterm, which is not what commonly happens. Secondly, they do not provide basic information for delivery (Gestational age at delivery, birth weight, birth weight centile). Thirdly, they do not provide their criteria for diagnosis of the problem (IUGR) but rather assume it was previously diagnosed.

**Authors**: Dear reviewer thank you very much for the comment. We have included hospital protocol for the diagnosis and follow up of growth restriction (Page 7, lines 135-148). Regarding the gestational age of term and preterm it might because what is presented is gestational age at delivery. With gestational age at delivery almost 50% are preterm and 50% are term. There is no statistical significant difference in terms of birthweight between two groups as shown in table 2.
