# Supplementary material for: Perinatal outcome of growth restricted fetuses with abnormal umbilical artery Doppler waveforms compared to growth restricted fetuses with normal umbilical artery Doppler waveforms at a tertiary referral hospital in urban Ethiopia
Source: PLoS One. 2020 Jun 18;15(6):e0234810. doi: 10.1371/journal.pone.0234810 (PMC7302535; doi:10.1371/journal.pone.0234810)
Supplement: Attachment — Submitted filename: Reviewer response May 26.docx [file pone.0234810.s003.docx]

May 24, 2020

To: PLOS ONE Editor in chief.

Dear Editor in chief.

We would like to thank the editor and reviewers for their thoughtful review of the manuscript. They raise important issues and their inputs are very helpful for improving the manuscript. We agree with almost all their comments and we have revised our manuscript accordingly. We respond below in detail to each of the editor comments. We hope that you find our responses satisfactory and that the manuscript is now acceptable for publication

Looking forward hearing from you soon

Sincerely,

Lemi B Tolu (MD, Assistant prof of obstetrics and gynecology).

Saint Paul’s Millennium Medical College(SPHMMC)

Department of Obstetrics and Gynecology

Addis Ababa, Ethiopia.

Email: [lemi.belay@gmail.com](mailto:lemi.belay@gmail.com)

**Dear editor**

Thanks for thoughtful review of the manuscript. Below is point by point response to raised concerns and how we changed the manuscript according to the comments.

1. Firstly, the manuscript has multiple typographical and grammatical errors and thus, a more thorough editing process is necessary. Please replace “doppler” by Doppler in all the manuscript.

**Authors**: Dear editor thank you very much, two authors independently revised the typos and grammar of the manuscript and much is changed.

2. Normal umbilical artery Doppler should be considered as values below the 95th centile, values below the 10th centile should not be considered abnormal. An international consensus exists for definition of abnormal UA Doppler by including only the pulsatility index and therefore, UA Doppler indices such as S/D ratio and RI should be excluded.

**Authors**: Dear editor thank you very much we have corrected definition (page 7, line 135). On which indices to use, as you said PI is most recommended. But still raised S/D or absent or reversed doppler are abnormal that’s why we included in the operational definition.

3. Perinatal death should include also those cases with intrauterine fetal demise

Authors: Dear editor thank you very much, we have corrected with all appreciation, (Page 8, lines 151,152)

4. In the abstract section, please include the p values between the study group comparisons.

**Authors:** Dear editor thank you, corrected (abstract section)

5. “On the 7th neonatal day, 129(97%) of normal and 29(78.4%) of abnormal umbilical artery doppler were alive whereas 4(3%) of normal and 9(24.3%) of abnormal umbilical artery Doppler studies ended in early neonatal death”. Please replace this paragraph by the proportion of perinatal death between the study groups.

**Authors**: Dear editor thank you very much for the feedback, we have corrected (page 2, line 35, page 10 line 233)

6. The frequency of adverse perinatal outcome (perinatal death, NICU admission, neonatal morbidity) should be adjusted by gestational age at birth) and all such outcomes (and not only NICU admission and neonatal death) should be also summarized in the abstract section and in the Results section.

**Authors**: Dear editor that’s very true, thanks a lot. Upon multiple regression only NICU admission, respiratory distress and perinatal death is associated with gestational age, that’s why we did for them. We have included in the abstract section as limitation of the study.

7. Please specify in the Methods section how was the UA Doppler evaluated, site and angle of insonation, number of included waveforms and ultrasound settings, etc. Were all ultrasound Doppler measurements performed at the day of delivery? Please specify.

Authors: Dear editor thank a lot. We have included what we use in the method section (Page 6 lines 102-104,114)
